# Effects of light attenuation on the sponge holobiont- implications for dredging management

**DOI:** 10.1038/srep39038

**Published:** 2016-12-13

**Authors:** Mari-Carmen Pineda, Brian Strehlow, Alan Duckworth, Jason Doyle, Ross Jones, Nicole S. Webster

**Affiliations:** 1 Australian Institute of Marine Science, Townsville, QLD, and Perth, WA, Australia; 2 Western Australian Marine Science Institution, Perth, WA, Australia; 3 Centre for Microscopy Characterisation and Analysis, UWA Oceans Institute, and School of Plant Biology: University of Western Australia, Crawley, WA, Australia

## Abstract

Dredging and natural sediment resuspension events can cause high levels of turbidity, reducing the amount of light available for photosynthetic benthic biota. To determine how marine sponges respond to light attenuation, five species were experimentally exposed to a range of light treatments. Tolerance thresholds and capacity for recovery varied markedly amongst species. Whilst light attenuation had no effect on the heterotrophic species *Stylissa flabelliformis* and *Ianthella basta,* the phototrophic species *Cliona orientalis* and *Carteriospongia foliascens* discoloured (bleached) over a 28 day exposure period to very low light (<0.8 mol photons m^−2^ d^−1^). In darkness, both species discoloured within a few days, concomitant with reduced fluorescence yields, chlorophyll concentrations and shifts in their associated microbiomes. The phototrophic species *Cymbastela coralliophila* was less impacted by light reduction. *C. orientalis* and *C. coralliophila* exhibited full recovery under normal light conditions, whilst *C. foliascens* did not recover and showed high levels of mortality. The light treatments used in the study are directly relevant to conditions that can occur *in situ* during dredging projects, indicating that light attenuation poses a risk to photosynthetic marine sponges. Examining benthic light levels over temporal scales would enable dredging proponents to be aware of conditions that could impact on sponge physiology.

Sponges are filter-feeding organisms with important ecosystem roles including habitat provision, seawater filtration, primary production and binding and/or erosion of the carbonate reef structure^1^. This diversity of functional roles means that changes to the abundance or composition of sponge assemblages is likely to significantly impact other reef organisms and overall ecosystem functioning^2^. Importantly, sponges host dense and diverse microbial symbionts that are intimately linked to their health, fitness and nutrition^3^,^4^. Many of these sponge symbionts are photosynthetic and in some cases they produce >50% of the energy requirements of the host^5^,^6^. In these mutually beneficial partnerships, the photosymbionts obtain energy from sunlight and provide the host with glucose, glycerol and amino acids and, in return, are provided with a protected environment and many of the compounds required for photosynthesis^7^,^8^,^9^,^10^. These sponges are described as ‘phototrophic’ or ‘mixotrophic’ depending on their degree of nutritional dependence from the symbiont primary production^6^,^11^.

Phototrophic sponges are common in shallow tropical reef environments world-wide, including the Mediterranean, Red Sea and the Caribbean^3^,^6^ and can comprise up to 80% of individuals and biomass in some coral reefs in the Indo-West Pacific and the Great Barrier Reef (GBR)^3^,^12^. In these regions phototrophic sponges have important ecological roles as net primary producers as well as in recycling organic carbon through shedding of cellular material which is rapidly consumed by detritivores^3^,^13^.

Due to their nutritional dependence on the products of photosynthesis, phototrophic sponges may be particularly sensitive to turbidity (water cloudiness) and light reduction caused by increased suspended sediments in the water column^14^,^15^,^16^,^17^. One cause of high turbidity is dredging of the sea bed and the subsequent disposal of the sediment at offshore dredge material placement sites^18^. Recent analyses of the effects of plumes from dredging have shown the marked effects the suspended sediments can have on benthic light levels^19^,^20^,^21^. Close to working dredges all benthic light can be extinguished during the daytime; however, a more common feature is very low ‘caliginous’ or daytime twilight periods, which can occur over extended periods (i.e., days to weeks)^19^. The high suspended sediment concentrations (SSCs), and the resulting light attenuation, decreases with increasing distance from dredging and understanding the impact of light reduction at different degrees is important for environmental impact assessments and managing dredging programs when underway.

The impacts of light reduction are likely to vary between sponge species, and this is likely to depend on the flexibility of their feeding strategy. For example, whilst the nutrition of some phototrophic sponges can be adversely affected by light reduction and a reduction of photosymbiont derived carbon, other species may have the ability to increase heterotrophic feeding rates to compensate for the decrease in photosynthetic yields^6^,^7^. Although cyanobacteria are the dominant photosymbiont taxa in most sponges^10^,^22^, dinoflagellates (i.e., *Symbiodinium* spp.) are also found in association with clionaid sponges^23^,^24^; and rhodophytes, chlorophytes and diatoms have been reported from a diverse range of sponges^22^,^25^,^26^. Therefore, responses to light attenuation may also differ depending on the symbiont composition within the host. Furthermore, highly specialised, host-specific symbionts often provide a greater benefit to their hosts than generalist symbionts^14^, and thus any negative effects from light attenuation will have a greater relative impact on those sponge species that host obligate photosymbionts.

Close to dredging, sponges will be exposed to low light conditions in combination with high SSCs and sedimentation^20^. This makes it difficult to identify cause-effect pathways, to identify potential bio-indicators and to establish the dose-response relationships needed for impact-prediction purposes. The effects of sedimentation on different sponge species, with a focus on sponge morphology, have been described previously^27^. In the current study, we examined the effects of light reduction alone on five abundant and geographically widespread sponge species^1^,^28^, spanning heterotrophic and phototrophic nutritional modes. In future studies, the effects of elevated SSC alone, sediment smothering alone and a combination of all dredging related factors will be described.

Using environmentally relevant exposure scenarios is essential for risk assessment purposes, and we used light reduction scenarios recently reported in several large-scale capital dredging projects on reefs in Western Australia^19^,^21^. The Daily Light Integral (DLI), which is the total photosynthetically active radiation (PAR) received each day as a function of light intensity and duration (mol photons m^−2^ d^−1^), was calculated over different running mean time periods from 1–30 days. For several shallow water sites (~6–9 m depth) located <1 Km from the dredge, the 5^th^ percentile (*P*_5_) of DLIs ranged from 1.3–2.1 mol photons m^−2^ d^−1^ before dredging, but was 0.4–0.7 mol photons m^−2^ d^−1^ during the dredging phase. The number of consecutive days of very low light (<0.8 mol photons m^−2^ d^−1^) ranged from 0–10 d before dredging, but from 11–25 d during the dredging phase. For near complete loss of all light, the worst case scenario during the baseline phase was 2 consecutive days increasing to 5 consecutive days during the dredging phase^19^.

In this study, a suite of different response variables were used to examine the effects of environmentally realistic light reduction over an extended period (30 d), with a particular focus on changes in sponge symbiosis.

## Results

### Host response (health)

All sponges survived the 28 d experimental treatments, with no observable partial mortality (tissue necrosis). Changes in colour were observed in two of the three phototrophic species. *Cliona orientalis* discoloured after 72 h in the 0 mol photons m^−2^ d^−1^ treatment, and milder discolouration occurred in some individuals in the 0.8 mol photons m^−2^ d^−1^ treatment (see Supplementary Fig. S1). Despite this rapid response, the colouration returned to normal when returned to natural light for the 14 d observational period. *Carteriospongia foliascens* also discoloured in the 0 mol photons m^−2^ d^−1^ treatment after 7 d, and whilst there was no partial mortality at the end of the experimental period (Supplementary Fig. S1), all discoloured individuals died during the observational period. The third phototrophic sponge species, *Cymbastela coralliophila,* and the heterotrophic species *Stylissa flabelliformis* and *Ianthella basta*, showed no signs of discolouration throughout the experimental or recovery periods (Supplementary Fig. S1).

### Host response (growth)

**Figure 1:**
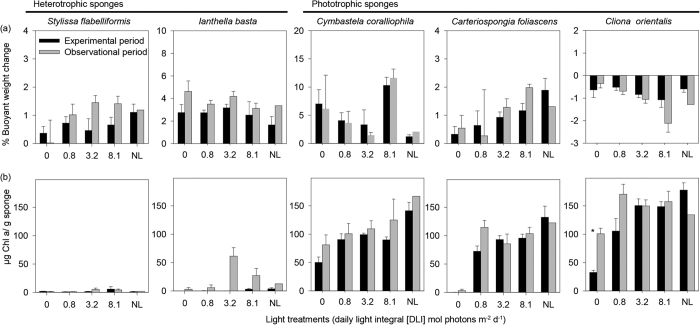
Physiological responses of sponges to light conditions. (**a**) Percentage buoyant weight change (note: y-axis scales differ between species), and (**b**) Chlorophyll a concentrations, for the five species, after the 28 d experimental period and 14 d observational period (mean ± SE). Asterisks show statistically significant differences between the experiment and recovery phase (T-tests: *P* < 0.05). Light treatments include 0, 0.8, 3.2, 8.1 mol photons m^−2^ d^−1^ and natural light (NL, 3.2–6.5 mol photons m^−2^ d^−1^).

*S. flabelliformis, I. basta, C. coralliophila* and *C. foliascens* grew, or gained weight, over the experimental period and 14 d observational period in almost all treatments (Fig. 1a). Some individuals of *C. foliascens* from the 0 and 0.8 mol photons m^−2^ d^−1^ treatments lost weight during the recovery phase. The buoyant weight of the bioeroding, encrusting sponge *C. orientalis* decreased across all treatments (Fig. 1a), reflecting active bioerosion of the coral substrate. The percentage change in buoyant weight from the start of the experimental period to the end of the observational period was not significantly different between treatments for any species except *C. orientalis* (Table 1A). *C. orientalis* exposed to the 0 and 0.8 mol photons m^−2^ d^−1^ treatments lost significantly less weight than individuals in the 8.1 mol photons m^−2^ d^−1^ treatment. Accordingly, the mass of excavated substrate per unit area (i.e., surface area) in *C. orientalis* was the lowest under the 0 mol photons m^−2^ d^−1^ treatment, although this was not statistically significant (ANOVA: *P* = 0.870).

No change in sponge surface area between treatments could be detected at the end of the experimental period for any species and, with the exception of *C. foliascens*, no significant differences in surface area were detected between treatments from the start of the experimental period until the end of the observational period (Table 1B). A significant reduction in surface area was observed for *C. foliascens* at the end of the observational period (consistent with partial mortality in some individuals exposed to the 0 mol photons m^−2^ d^−1^ treatment, see Supplementary Fig. S2). Surface area could not be measured in *C. orientalis* which grows deeper into the coral substrate whilst still maintaining a constant surface area due to its bioeroding nature.

### Microbial community response (pigment analyses)

Chlorophyll a concentrations of the phototrophic species *C. coralliophila, C. foliascens* and *C. orientalis* were typically higher than ~100 μg Chl a/g sponge, and concentrations in the heterotrophic species *S. flabelliformis* and *I. basta* were negligible (overall, < 10 μg Chl a/g sponge) (Fig. 1b). The only exception to this occurred in the 3.2 and 8.1 mol photons m^−2^ s^−1^ treatments for *I. basta* during the observational period, in which an algal bloom corresponded with the time of sampling (Fig. 1b). In both heterotrophic species, no significant difference in Chl a was detected between light treatments after the experimental or observational period (T-tests: *P* > 0.1) (Table 1C). The Chl a concentration of the phototrophic species under natural light was similar to the freshly collected field samples and t = 0 controls (ANOVA: *P* > 0.05). There were significant differences between the light treatments in the phototrophic species, and the highest Chl a concentration was observed in the natural light controls, followed by the 8.1, 3.2, 0.8 and 0 mol photons m^−2^ d^−1^ treatments (Fig. 1b, Table 1C). In the lower light treatments (0 and 0.8 mol photons m^−2^ d^−1^) there was an overall increase in Chl a concentration after the observational period in natural light (Fig. 1b).

Total chlorophyll was highly correlated to Chl a in the three phototrophic species (R^2^ = 0.834, 0.992, 0.994 for *C. coralliophila, C. foliascens* and *C. orientalis*, respectively, *P* < 0.001). However, total chlorophyll was also marginally correlated to Chl d in *C. coralliophila* (R^2^ = 0.305, *P* = 0.07), and highly correlated to Chl d in *C. foliascens* (R^2^ = 0.847, *P* < 0.001), and to Chl c in *C. orientalis* (R^2^ = 0.906, *P* < 0.001), consistent with the abundance of cyanobacteria in *C. foliascens* and *C. coralliophila* and *Symbiodinium* sp. in *C. orientalis*^23^,^24^. Accordingly, significantly lower values of Chl d and c were retrieved from *C. foliascens* and *C. orientalis*, respectively, in the 0 mol photons m^−2^ d^−1^ treatment (ANOVA: *P* < 0.001), while no signficant differences in Chl d were observed in *C. coralliophila* (ANOVA: *P* < 0.681).

Ultra Performance Liquid Chromatography (UPLC) analysis detected Chl a, Chl b, Chl c, Pheophytin a and six Carotenoids (i.e., Peridinin, Neoxanthin, Violaxanthin, Diadinoxanthin, Dinoxanthin and Zeaxanthin) with distinct, species-specific pigment profiles. Non-metric Multi-Dimensional Scaling (nMDS) analysis of the normalized spectrophotometry data and UPLC data showed that both approaches resulted in comparable sample groupings (see Supplementary Fig. S3) and were significantly correlated (R^2^ ≥ 0.8 and *P* < 0.001 in all cases). *C. foliascens* and *C. coralliophila* samples showed greater similarity in pigment profiles than *C. orientalis*, consistent with the presence of cyanobacterial photosymbionts in the former species and *Symbiodinium* sp. in the latter (Supplementary Fig. S3).

Non-metric MDS ordination analysis revealed that most sponge samples in the zero light treatment grouped closely together irrespective of species (Supplementary Fig. S3). PERMANOVA confirmed significant differences between species and treatments for both methods (Table 2). Subsequent pair-wise tests showed significant differences between samples in the dark and the natural light controls in all cases (Table 2) and *C. foliascens* samples in the 0.8 mol photons m^−2^ d^−1^ treatment differed from samples in the control and dark treatments.

Zeaxanthin is a pigment commonly related to the presence of Cyanobacteria^29^. Accordingly, it was significantly higher in *C. coralliophila* and *C. foliascens* than in *C. orientalis* (ANOVA: *P* = 0.002), while no significant differences between *C. coralliophila* and *C. foliascens* were detected (ANOVA: *P* > 0.05). This pigment was lowest in samples of *C. coralliophila* and *C. foliascens* in the zero light treatment (ANOVA: *P* = 0.103, < 0.005, respectively), followed by samples in the 0.8 mol photons m^−2^ s^−1^ and the natural light treatments. While Zeaxanthin concentration in *C. orientalis* was not significantly different between treatments (ANOVA: *P* = 0.469), concentrations dropped to zero in samples in the 0 mol photons m^−2^ d^−1^ treatment. On the other hand, Peridinin, which is an additional light harvesting pigment common in *Symbiodinium* sp., was exclusively present in *C. orientalis*. Whilst Peridinin was lowest in samples exposed to zero light, differences between treatments were not significant (ANOVA: *P* = 0.085).

Additional correlations were performed to determine which pigments best explained trends in sponge growth (i.e., based in buoyant weights at the end of the experimental period) within each phototrophic species. No significant correlations between any pigment and buoyant weight were detected in *C. coralliophila* and *C. orientalis*. However, growth rates were significantly correlated to all pigments in *C. foliascens* (i.e., Chl a, Chl d, Pheophytin, Zeaxanthin, Carotenoids and Total Chl; R^2^ > 0.7, *P* < 0.05), corroborating the higher degree of *C. foliascens* dependence on phototrophic nutrition.

### Microbial community response (chlorophyll fluorescence)

**Figure 2:**
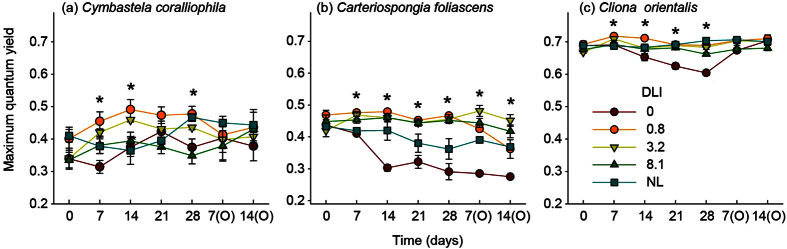
Temporal evolution of fluorescence levels. Mean values (±SE) of fluorescence (maximum quantum yield) for the three phototrophic species per light treatment 0, 0.8, 3.2, 8.1 mol photons m^−2^ d^−1^ and natural light (NL, 3.2–6.5 mol photons m^−2^ d^−1^). Measurements were taken at the start of the experiment and then weekly until the end of the experimental and observational (O) periods. Asterisks show statistically significant differences among treatments (ANOVA: *P* < 0.05).

Chlorophyll fluorescence was not significantly different between treatments at the start of the experiment for any species (Fig. 2). After 7 d, maximum quantum yields varied significantly among light intensities for all of the phototrophic species. Overall, individuals exposed to the 0.8 and 3.2 mol photons m^−2^ d^−1^ treatments exhibited increased photosynthetic efficiency during the first weeks (Fig. 2). Subsequently, both *C. orientalis* and *C. foliascens* showed a significant decrease in chlorophyll fluorescence in the 0 mol photons m^−2^ d^−1^ treatment. With the exception of *C. foliascens* in the zero light treatment, which suffered 100% mortality, fluorescence values of all other samples returned to normal during the observational phase under natural light conditions.

### Microbial community response (microbiome)

**Figure 3:**
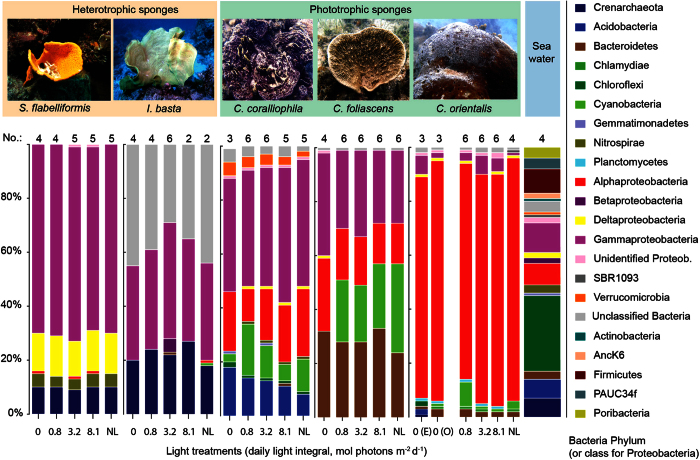
Phylum-level bar chart. Average relative abundance of each bacteria phylum (and class for Proteobacteria) for each species and light treatment (0, 0.8, 3.2, 8.1 mol photons m^−2^ d^−1^ and natural light: NL, 3.2–6.5 mol photons m^−2^ d^−1^). Only OTUs representing greater than 1% of the overall community were included. When significant differences in time were detected, samples from experimental (E) and observational (O) periods were plotted separately (i.e., 0 mol photons m^−2^ d^−1^ in C. *orientalis*, Table 3).

A total of 13,240,437 high quality 16 S rRNA gene amplicon sequences were recovered from the 5 sponge species (n = 168 individual samples) and 5 seawater samples (environmental controls). Following quality filtering, 25 samples with low (<4906) read numbers were eliminated from the dataset which included 6 from *S. flabelliformis*, 10 from *I. basta*, 8 from *C. coralliophila*, and 1 seawater environmental control. The remaining samples were sub sampled down to the lowest read number (6587). Each sponge species maintained a unique microbial community (Table 3A, Fig. 3, see also Supplementary Fig. S4) that was distinct from the seawater microbiome (Table 3B, Fig. 3, Supplementary Fig. S4). Aquarium acclimation (for ~6 weeks) did not affect the sponge-associated microbial community of any species, as evidenced by the microbiome similarity of the field controls and the t = 0 experimental samples (Table 3A). Overall, the microbial community of the heterotrophic species *S. flabelliformis* and *I. basta*, and the phototrophic species *C. coralliophila*, was consistent amongst all sampling times (Table 3A). Highly significant differences in microbial community composition were observed in the phototrophic species *C. foliascens* and *C. orientalis* over time (i.e., from t = 0 until the end of the experimental period) (Table 3A).

**Figure 4:**
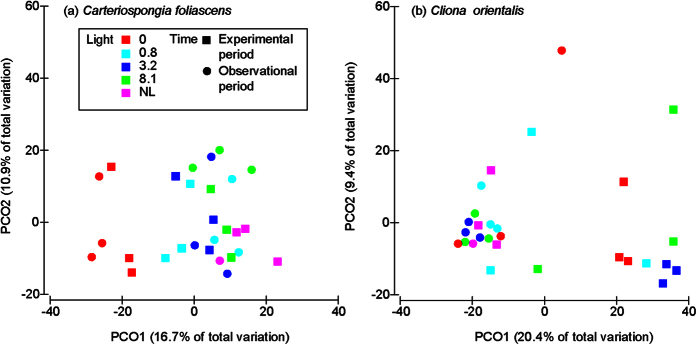
PCO analyses of *C. foliascens and C. orientalis*. Principal coordinate analysis plots for *C. foliascens* and *C. orientalis* which showed significant differences based on light treatment or time according to PERMANOVA analysis (Table 3C). Square symbols represent samples at the end of 28 d experimental period, circles are samples at the end of the observational period and colours indicate the 5 light treatments (0, 0.8, 3.2, 8.1 mol photons m^−2^ d^−1^ and natural light: NL, 3.2–6.5 mol photons m^−2^ d^−1^).

Microbial community composition did not differ between light treatments for either heterotrophic species or the phototrophic *C. coralliophila*, but differed between treatments in the phototrophic *C. foliascens* and *C. orientalis* (Table 3C, Fig. 3). Whilst no significant microbial shifts were detected in *C. orientalis* under different light treatments when all sample times were compared together (experimental and observational periods), differences were evident within sampling times when analysed independently (particularly in the zero light treatment) (Table 3C, Figs 3 and 4).

**Figure 5:**
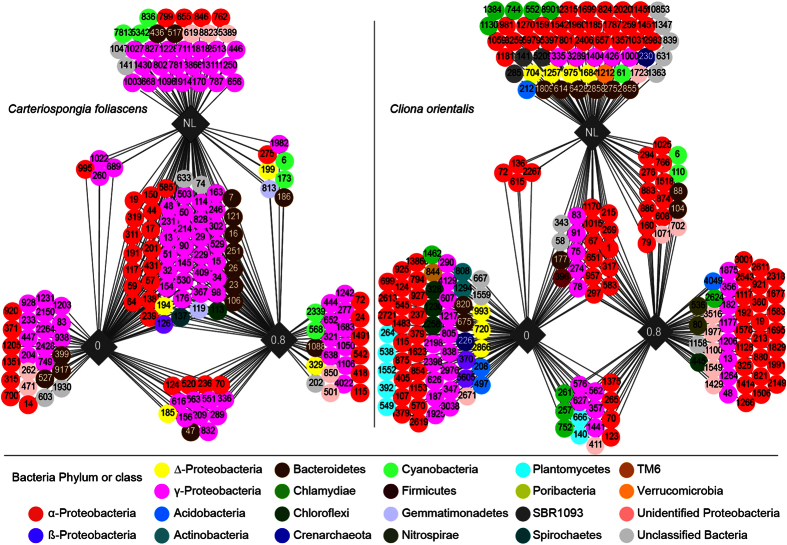
Cytoscape networks of *C. foliascens* and *C. orientalis* microbiome. Networks of the microbiome community on 3 light treatments (0, 0.8 and natural light (3.2−6.5 mol photons m^−2^ d^−1^)) for the phototrophic species *C. foliascens* and *C. orientalis*. Circles correspond to different OTUs (with OTU numbers) and colours relate to their Phylum or Class level in the case of *Proteobacteria*. Only samples at the end of the 28 d experimental period were used (n = 3 per treatment).

At the Phylum level, the microbiome of *C. coralliophila* was primarily comprised of *Alpha* and *Gammaproteobacteria, Acidobacteria* and *Cyanobacteria* with only a small decrease in the relative abundance of *Cyanobacteria* occurring in *C. coralliophila* in the absence of light (Fig. 3). In contrast, the microbial community of *C. foliascens* in the zero light treatment showed a complete loss of *Cyanobacteria* and no recovery after the observational period (Table 3C, Figs 3 and 4). The prokaryotic microbiome of *C. orientalis* was primarily comprised of *Alpha* and *Gammaproteobacteria* and while minor representation of *Cyanobacteria* was evident in *C. orientalis* from most light treatments, this taxa was absent in the zero light treatment (Figs 3, 5 and Table S1). However, *C. orientalis* was able to re-establish its native microbiome after recovery under natural light (Fig. 4).

Network analyses of the 100 most discriminatory operational taxonomic units (OTUs) were performed on *C. foliascens* and *C. orientalis*, for the 0, 0.8 mol photons m^−2^ d^−1^ and natural light treatments. The microbial community in *C. foliascens* was comprised of a large core microbiome (i.e., the component shared between samples in all treatments) that was unaffected by light treatment (Fig. 5). However, the three dominant *Cyanobacteria* OTUs (836, 5342 and 7813) were highly sensitive to light attenuation, being exclusively present in samples from the natural light treatment only. In contrast, *Cyanobacteria* OTUs 6 and 173 could tolerate the 0.8 mol photons m^−2^ d^−1^ treatment, but disappeared in the absence of light. This species also appears to shift its photosymbiont community under reduced light, with samples at the 0.8 mol photons m^−2^ d^−1^ acquiring novel *Cyanobacteria* OTUs 568 and 2339 which were exclusively present in *C. foliascens* from this treatment. Network analysis of the *C. orientalis* microbiome revealed a much smaller core community, and a large number of OTUs that were highly specific to samples in each light treatment (Fig. 5). As in *C. foliascens*, some *Cyanobacteria* OTUs (6 and 110) persisted under the natural light and 0.8 mol photons m^−2^ d^−1^ treatments, but disappeared in the zero light treatment, while *Cyanobacteria* OTU 61 was highly light sensitive and persisted in *C. orientalis* only under natural light conditions. Apart from the *Cyanobacteria*, the light sensitive community (i.e., taxa which were exclusively present in samples under natural light) included representatives of the Phyla *Proteobacteria, Bacteroidetes, Chlamydiae, Verrucomicrobia* and *Acidobacteria* as well as the candidate phylum SBR1093 and the *Archaea.* In contrast, in the zero light treatment, *C. orientalis* was colonised by novel OTUs within the *Actinobacteria, Planctomycetes, Spirochaetes, Chloroflexi* and additional OTUs affiliated to the *Proteobacteria* and *Acidobacteria*.

## Discussion

Prolonged periods of low light caused by natural sediment resuspension events or dredging activities have the potential to adversely affect phototrophic sponges, although tolerance thresholds and capacity for recovery varied markedly in this study amongst sponge species. In contrast, heterotrophic sponge species appear unaffected by light attenuation as indicated by highly stable microbiomes, similar growth rates across all treatments and no visible signs of stress, including pigmentation changes or partial mortality.

The photosynthetic bioeroding sponge *C. orientalis* discoloured within as little as 7 d in darkness and exhibited minor discolouration after 28 d in the highly light attenuated treatment (0.8 mol photons m^−2^ d^−1^). These visible effects were accompanied by a significant decrease in chlorophyll a concentrations and changes in maximum quantum yields. This discolouration was ‘sponge bleaching’^30^, caused by loss of all *Cyanobacteria* and a marked reduction in the concentration of Chlorophyll a-containing *Symbiodinium* sp. Although bleaching occurred rapidly, *C. orientalis* still maintained quite high fluorescence values and also showed a rapid recovery of pigmentation on return to natural light conditions. *Cliona varians*, a congeneric species from the Caribbean, can also rapidly recover its native symbionts (within 2 weeks) following 8 months of exposure to total darkness^31^. Histological analysis of the *C. orientalis* tissues revealed the presence of some *Symbiodinium* deep in the tissue even after 28 d in darkness. Bioeroding *Cliona* sponges appear to be able to cope with extended periods of light reduction by retracting tissue and symbionts deep into the host coral skeleton^32^. This behavioural response could also have contributed to at least some of the discolouration, similar to effects observed with scleractinian corals where extreme retraction of the *Symbiodinium* spp. containing tissues during sub-aerial exposure can cause them to pale. This bleaching in corals is rapidly reversible (within hours) on return to more benign conditions.

It is not clear how the *Symbiodinium* sp. in *C. orientalis* could survive such periods of complete darkness. In culture, *Symbiodinium* spp. have been reported to undertake heterotrophic feeding^33^, although it remains to be determined whether this can occur *in hospite*. Nevertheless, the remnant symbiont population is significant, as the rapid division of this population can mean a relatively quick recovery from bleaching on return to the more favourable conditions.

*C. foliascens* survived the 28 d exposure to complete darkness, but underwent a loss of photosymbionts, a large shift in the microbiome and subsequently died in the observational period on return to natural light conditions. In contrast, the phototrophic *C. coralliophila* showed a high tolerance to reduced light as evidenced by only minor bleaching, slight reductions in chlorophyll a, minor changes in quantum yield values and a decrease in the relative abundance of *Cyanobacteria* in darkness. No significant changes were observed in the overall microbiome and complete recovery was observed within two weeks under natural light conditions.

Light is a critical factor controlling the growth and population demographics of phototrophic sponges as photosynthetic productivity from symbiotic microbes can provide between 48–80% of a sponge’s energy requirements^3^. Reduced light availability is therefore linked to energy depletion and associated mass reduction, as observed here for *C. foliascens* and as previously reported for other sponge species^12^,^14^. Light is also considered an important factor influencing excavation rates by bioeroding species^1^,^24^, and although some *Symbiodinium* sp. remained deep in the tissue even after four weeks without light, *C. orientalis* exposed to low light intensity also appeared to have reduced bioerosion capability, as inferred by a significantly smaller reduction in buoyant weight compared to control samples.

Although most phototrophic sponge species rely primarily on symbiotic relationships for their energy needs^6^,^12^, some have the ability to activate their heterotrophic metabolism under certain conditions. This switch to heterotrophy would enable sponges to survive under reduced light conditions, as has been reported in *Petrosia ficiformis* (Poiret, 1789), *Aplysina fulva* (Pallas, 1766) and *Neopetrosia subtriangularis* (Duchassaing, 1850)^6^,^7^. The same switching has also been observed in photosymbiotic corals during low light associated with natural turbidity events^34^.

The different responses of the three phototrophic sponges under low light conditions may be related to different degrees of dependency on the photosynthetic symbionts. For instance, *Phyllospongia lamellosa* (Esper, 1794), a foliaceous phototrophic sponge closely related to *C. foliascens*^35^, relies on photosynthesis for the majority of its respiratory carbon requirements^12^. In *C. foliascens* the symbiosis is highly specialized and largely driven by a *Synechococcus* sp. (OTU 6)^36^. The strong correlation between photopigments and growth rates in *C. foliascens* provides further support for a high degree of nutritional dependence on photosymbionts. In contrast, *C. coralliophila* tolerated the 4-week light attenuation and its growth rate showed no correlation with any pigment, suggesting that it may have the ability to increase its heterotrophic feeding rates when ambient light conditions change and photosynthetic activity of symbionts is reduced. However, longer-term experiments are still required as a 90 day light attenuation study on a congeneric species, *Cymbastela concentrica* (Lendenfeld, 1887), resulted in bleaching, necrosis, and significant reductions in growth, reproductive status and concentration of chlorophyll a^15^.

While many sponge species host highly stable microbiomes irrespective of environment^37^,^38^,^39^,^40^, recent research has also indicated that shifts in the sponge-associated microbial community may confer a mechanism for local host acclimation to different environmental conditions^41^. The large shifts in the microbiome of *C. foliascens* and *C. orientalis* under low light conditions, including the appearance of novel photosynthetic microorganisms under highly light attenuated conditions (DLI of 0.8 mol photons m^−2^ d^−1^), may indicate an attempt at symbiont shuffling to a community better able to function under reduced irradiance. The rapid microbiome recovery of both *C. orientalis* and *C. coralliophila* under natural light further indicates significant flexibility in symbiosis in these species. However, while some species may have the ability to successfully alter the composition of their microbial community under different environmental conditions^42^, an extensive body of literature also highlights that species with intimate and/or potentially obligate symbioses can be adversely impacted by disruption of the microbiome^43^,^44^ or loss of symbiotic function^45^. The rapid deterioration in health of *C. foliascens* following the microbiome shift is consistent with its intimate reliance on a highly specialised microbial community.

The levels of light reduction attained close to dredging activities were recently described in a large-scale capital project on reefs in Western Australia^19^,^21^. The light treatments used in the present study are clearly relevant to conditions that can occur during dredging, showing that light attenuation is not only a hazard to photosynthetic sponges but also a risk in close proximity to dredging operations^46^. This study examined the specific effects of light reduction on sponges, with the combined effects of elevated SSCs and light attenuation being investigated in subsequent experiments. Nevertheless, based on light reduction alone, the rapid bleaching response observed in *C. foliascens* (irreversible) and *C. orientalis* (reversible) suggests that discolouration of sponges would be an effective bioindicator during dredging operations, as long as natural variations in colour are taken into consideration. The results of this study could be used in conjunction with water quality monitoring programs to alert dredging proponents to levels of light reduction that, if continued, could result in harm to phototrophic sponges. In combination with sediment plume models and estimates of the light attenuation by the suspended sediment, these results could be used to make predictions of the likely effects of dredging (i.e., at the EIA stage). By modelling different dredging scenarios (i.e., volume of material dredged, tidal phase, over flow options, etc.) the information could also be used to identify optimal dredging scenarios that minimise the likelihood and extent of impact. Benthic light availability should be considered over multiple different time periods (from days to weeks) allowing dredging proponents to be aware of, or alerted to, conditions that could potentially impact sponge physiology.

## Methods

### Sample collection

To facilitate a response comparison across nutritional modes, the study was conducted with the phototrophic sponges *Cymbastela coralliophila* (Hooper & Berquist 1992), *Carteriospongia foliascens* (Pallas, 1766) and *Cliona orientalis* (Thiele, 1900), and the heterotrophic sponges *Stylissa flabelliformis* (Hentschel, 1912) and *Ianthella basta* (Pallas, 1976)^12^,^23^,^27^,^47^,^48^. All sponges were collected from 3–15 m depth from either the Palm Islands or Davies Reef, Great Barrier Reef (GBR) (see Supplementary Table S2). *C. orientalis* is an encrusting sponge that bioerodes coral, hence cores of sponge on top of dead coral substrate where chiselled from *Porites* sp. colonies. Sponges were cut into similar sized explants (~10 × 10 cm), and placed in natural light acclimation tanks with flow-through seawater for up to 6 weeks until they had completely healed.

### Experimental Set up

All experiments were performed in the National Sea Simulator (SeaSim) at the Australian Institute of Marine Science (AIMS, Townsville). Tests were conducted in 50 L acrylic tanks supplied with a continous inflow of 5 μm filtered seawater at a rate of 833 mL min^−1^. The high inflow rates ensured one complete turnover of the tanks per hour of partially filtered seawater, which ensured that sponges received sufficient particulate food including microoganisms. Experiments were conducted in a constant environment room with water temperature set to 29 °C, representing the temperature at the time of sponge collection. Sponges were exposed to 5 different light treatments selected to represent different levels of light attenuation in a dredging plume^19^,^49^. Tanks were illuminated on a 12:12 h L:D cycle, with a light regime designed to simulate daily conditions on the reef. Each day there was a 3 hour morning period (from 06:00–09:00) of gradually increasing light from darkness to a maximum instantaneous light level of 25, 100 and 250 μmol photons m^−2^ s^−1^ for each of the 3 treatments, followed by a 3 h afternoon period (from 15:00–18:00) of gradually decreasing light until full darkness. A natural light control (~150–300 μmol photons m^−2^ s^−1^ at noon) was also used, and the last treatment was complete darkness. Factoring in the increase and decrease in light levels and maximum instantaneous light level during the middle of the day, the daily light integrals (DLIs) were 0, 0.8, 3.2 and 8.1 mol photons m^−2^ d^−1^ respectively, and 3.2–6.5 mol photons m^−2^ d^−1^ for the natural light control, depending on weather conditions. The light attenuation experiment was conducted for a 28 d ‘experimental’ period, followed by a 14 d ‘observational’ (i.e., recovery) period under natural light. There were 3 tanks per treatment, each with 2 sponge replicates per species (i.e., 6 replicates of each species per treatment).

### Studied parameters

The effect of the different light treatments on the sponge holobiont was determined using a suite of response variables, with a particular focus on changes in sponge photosymbionts and composition of the sponge microbial community. For each species, 3 extra individuals were collected as field controls, and immediately processed *in situ* (see below) in order to determine any potential effects of the handling and experimental enclosures. At the start of the experiment, 3 additional individuals per species were sampled (t = 0 controls) to obtain baseline data on sponge health. Unless otherwise stated, statistical analyses were performed using the software SigmaPlot v.11.0 (Systat Software Inc.).

### Sponge growth

To obtain a proxy for sponge growth, initial and final weights were measured for all individuals using a buoyant weight scale (±0.001 g) at a constant temperature of 29 °C. Among a selection of methods to measure growth, buoyant weights were used to prevent potential blockages from air exposure, to allow subsequent tissue sampling and because they are good estimators of sponge growth across environmental gradients^50^. In the encrusting *C. orientalis*, buoyant weights indicate the net change between the weight of tissue (i.e., mostly spicules) and changes in the CaCO_3_ substrate caused by its bioeroding activity. Hence, weights in *C. orientalis* are not comparable with other sponge species. The mass of excavated substrate per unit area was also calculated in *C. orientalis* using surface area based on images (see below). However, due to the irregularity in shape of some of the replicates, these values are an estimation rather than exact size values. The percentage weight change between day 0 and the end of the recovery phase was assessed for each species separately using one-way analysis of variance (ANOVA) with treatment as the fixed factor. The mass of excavated substrate per unit area between day 0 and the end of the experimental phase was also assessed for *C. orientalis* using one-way ANOVA and treatment as fixed factor.

A 2-dimensional proxy for partial mortality (tissue necrosis), size change (i.e., surface area), and colour was recorded weekly using a digital camera with underwater housing (Canon PowerShot SX50-HS) and image analysis software (Image J^51^). Images were always taken under maximum instantaneous light levels (9.00–15.00 h) to eliminate any possible diurnal variation in colour. Changes in percent surface area after the experimental and observational periods were studied for each species separately using one-way ANOVA with treatment as the fixed factor. In any instances where homogeneity of variances and normality were not evident, a Kruskal-Wallis one-way ANOVA on ranks was performed.

### Pigment analyses

Pigment analyses were performed spectrophotometrically on the field controls, on the sponges collected at t = 0 and at the end of the experimental and observational periods. Two 1 × 0.5 × 0.5 cm pieces of tissue incorporating pinacoderm and mesohyl regions were excised per individual, placed immediately in liquid nitrogen and stored at −80 °C until pigment and symbiont analysis. Pigments were extracted from a weighed piece of sponge using 95% ethanol and a Bead Beater (Bio Spec Products Inc., Bartlesville, USA)^27^. Absorbance at 470, 632, 649, 665, 696 and 750 nm was measured using a Power Wave Microplate Scanning Spectrophotometer (BIO-TEK® Instruments Inc., Vermont USA). Chlorophyll a, b, c and d, and total carotenoids were calculated using the equations provided in refs 52,53 and standardized to sponge wet weight.

The concentration of chlorophyll a was used as a proxy for changes in photosymbiont health/activity (i.e., bleaching) due to light attenuation^3^. Changes in chlorophyll a concentration during the experimental period were assessed for each species separately using a one-way ANOVA with treatment as the fixed factor. In any instances where homogeneity of variances and normality were not evident, a Kruskal-Wallis one-way ANOVA on ranks was performed. Differences between chlorophyll a at the end of the experimental and observational periods were assessed with a t-test for each treatment and species, separately. Photopigments were additionally measured in a selection of samples (phototrophic species from the 0 and 0.8 mol photons m^−2^ d^−1^ and natural light treatments, n = 27) by Ultra Performance Liquid Chromatography (UPLC) to determine the type and quantity of pigments present^54^.

All pigments retrieved by spectrophotometry and UPLC were used to build resemblance matrices based on normalized data. Non-metric Multi-Dimensional Scaling (nMDS) plots were created for each method separately using Euclidean distances. Two factors were determined (species, light treatment) and examined by PERMANOVA (Permutational multivariate ANOVA based on distances). Analyses were performed using PRIMER 6 (Primer-E Ltd, UK).

### Chlorophyll fluorescence

Changes in photosynthetic capacity (maximum quantum yield [Fv/Fm]) of the sponge’s phototrophic symbionts were measured with a Diving-PAM (pulse amplitude modulation) chlorophyll fluorometer (Heinz Walz GmbH, Effeltrich, Germany). Chlorophyll fluorescence measurements were obtained 10 mm from the sponge tissue by placing a 6 mm fibre-optic probe perpendicular to the surface. Initial fluorescence was determined using a pulse-modulated red measuring light (655 nm, 0.15 μmol photons m^−2^ s^−1^ at 0.6 kHz). Three measures (maximum quantum yield) were obtained weekly on dark adapted samples (30 min) for the three phototrophic species (i.e., *C. coralliophila, C. foliascens* and *C. orientalis*) throughout the experimental and observational periods. One-way ANOVAs were performed to test whether treatment had any effect on chlorophyll fluorescence of the sponges at any given time. The effect of time within every treatment and species was also assessed with a one-way ANOVA.

### Microbial community analysis

The composition of the sponges microbial community was assessed using Illumina amplicon sequencing of the 16 S rRNA gene. Samples were collected at the end of the experimental and observation period, immediately frozen in liquid nitrogen and stored at −80 °C. Water samples were simultaneously collected from each tank to facilitate a direct comparison with microbes present in the surrounding environment. DNA was extracted from ~0.25 g of sponge tissue using the PowerSoil®-htp 96 Well Soil DNA Isolation Kit (MoBio Laboratories, Carlsbad, CA) according to standard protocols (http://press.igsb.anl.gov/earthmicrobiome/emp-standard-protocols/dna-extraction-protocol/). Microbial communities in seawater were collected by passing 1 L of seawater through 0.2 μm Sterivex filters and DNA was extracted from the filters as previously described^55^. Aliquots of the extracted DNA were shipped to the University of Colorado, (Boulder, Colorado, USA) for sequencing using standard Earth Microbiome Project (http://www.earthmicrobiome.org/emp-standard-protocols/16s/) protocols. Briefly, the V4 region of the 16 S rRNA gene was amplified using the primer 515 f – 806r and sequenced using the HiSeq2500 platform (Illumina). Processed sequences and meta-data are available via the following portal (http://qiita.microbio.me/) under study number 10533.

Amplicon sequence data was processed in Mothur v.1.35.1^56^. Firstly, quality-filtered, demultiplexed fastq sequences were trimmed according to quality and processed as per^57^. Only sequences that aligned to the expected position were kept. Aligned reads were reduced to non-redundant sequences and chimeric sequences were detected using Uchime^58^. Aligned sequences were phylogenetically classified based on the Ribosomal Database Project (RDP) reference file v.14^59^, and all undersigned sequences removed (taxon = Chloroplast-Mitochondria-unkown-Eukaryota). Pairwise distances between aligned sequences were calculated and used for clustering. Operational taxonomic units (OTUs) were retrieved based on the distance among the clustered sequences and were further classified based on the Greengenes taxonomy^60^.

Pivot tables were used to condense tables by phylum and class for graphical interpretation. OTU data was normalised to account for sampling depth and then square-root transformed to reduce the effect of abundant OTUs. Bray-Curtis distance matrices were constructed to examine additional patterns of community structure and visualised using non-metric multidimensional plots (nMDS) and principal coordinate analyses (PCO). Permutational analysis of variance (PERMANOVA, using 9,999 permutations) was used to determine significant differences in microbial communities based on source (sponge versus environmental control), time of sampling (field controls, t = 0, experimental and observational period), and all five light treatments. All multidimensional statistical analyses were performed in PRIMER 6. Similarity Percentage (SIMPER) analysis was used to determine the OTUs that contribute to the differences between the natural light, 0.8 and 0 mol photons m^−2^ d^−1^ treatments) for each phototrphic species, separetely. The 100 OTUs with the most discriminating power from the SIMPER analysis were used to create networks on Cytoscape 3.2.0 (www.cytoscape.org)^61^.

## Additional Information

**How to cite this article**: Pineda, M.-C. *et al*. Effects of light attenuation on the sponge holobiont- implications for dredging management. *Sci. Rep.* **6**, 39038; doi: 10.1038/srep39038 (2016).

**Publisher's note:** Springer Nature remains neutral with regard to jurisdictional claims in published maps and institutional affiliations.

## Supplementary Material

Supporting Online Material

Supporting Online Dataset

## Figures and Tables

**Table 1:** ANOVA examining the effect of treatment on A) Total change in sponge buoyant weight at the end of the observational phase, B) Changes in surface area for each species at the end of experimental and observational periods (surface area was not considered a relevant metric for growth of *C. orientalis*), and C) Chl a concentration at the end of the 28 d experimental period.

Source	*df*	*Stylissa flabelliformis*		*Ianthella basta*		*Cymbastela coralliophila*		*Carteriospongia foliascens*		*Cliona orientalis*	
		*F*	*P*	*F*	*P*	*F*	*P*	*F*	*P*	*F*	*P*
(A) Buoyant weight at the end of the observational phase											
Treatment	3	1.923	0.204	1.377	0.318	1.902	0.208	0.691	0.586	9.506	0.005
Error	8										
Significant Pairwise Multiple Comparisons (Holm-Sidak)										0,0.8,3.2 > 8.1	
(B) Surface area at the end of the experimental and observational phases											
Experimental phase											
Treatment	4	2.271	0.093	1.199	0.338	0.310	0.869	1.695	0.186		
Error	23										
Observational phase											
Treatment	3	1.789	0.227	1.330	0.331	0.943	0.464	4.186	0.047		
Error	8										
Significant Pairwise Multiple Comparisons (SNK)								8.1, NL, 3.2 > 0			
(C) Chl a at the end of the experimental phase											
Treatment	6	1.792	0.173	1.472	0.257	6.072	0.003	31.163	< 0.001	17.271	< 0.001
Error	14										
Significant Pairwise Multiple Comparisons (SNK)						NL,F, T0 > 0		T0,NL,F > 8.1,3.2,0.8 > 0		NL,F,8.1,3.2,0.8 > 0	

Holm-Sidak and Student Newman-Keuls (SNK) tests have been performed for significant pairwise multiple comparisons. In pair-wise tests, F: field control; t = 0: time 0 control; 0, 0.8, 3.2 and 8.1 mol photons m^−2^ d^−1^ treatments and natural light controls (NL).

**Table 2:** Two-way PERMANOVA of pigment data with species and treatment as factors, for A) Spectrophotometry and B) UPLC analyses.

Source	*df*	MS	Pseudo-*F*	*P* (perm)
A) Spectrophotometry				
Species	2	85.707	13.234	0.001
Treatments	2	47.827	7.3848	0.002
Sp × Treatm.	4	14.589	2.2526	0.019
Residuals	18	6.4765		
Pair-wise Tests				
CYM: D ≠ C (*P* = 0.022); CAR: L ≠ D ≠ C (*P* < 0.05); CLI: D ≠ C (*P* = 0.046)				
B) UPLC				
Species	2	27.666	20.451	0.001
Treatments	2	29.407	21.738	0.001
Sp × Treatm.	4	4.3757	3.2345	0.003
Residuals	18	1.3528		
Pair-wise Tests				
CYM: L, D ≠ C (*P* < 0.05); CAR: L ≠ D ≠ C (*P* < 0.05); CLI: L, C ≠ D (*P* < 0.05)				

In pair-wise tests, D: Dark (0 mol photons m^−2^ d^−1^), L: low light (0.8 mol photons m^−2^ d^−1^) and C: natural light controls (CLI for *C. orientalis*, CAR for *C. foliascens* and CYM for *C. coralliophila*).

**Table 3:** PERMANOVA analyses of the microbiome community with A) species and time as factors, B) source as factor (sponge host vs. environmental control) and, C) light treatment and time (nested to light) as fixed factors for all five sponge species.

Source	*df*	MS	Pseudo-*F*	*P* (perm)
A)				
Species	4	58196	40.879	0.001
Time	3	3161.1	2.2205	0.001
Species x Time	11	2291.3	1.6095	0.001
Residuals	124	1423.6		
Pair-wise Tests				
STY: F ≠ E, O (*P* < 0.005); IAN: F ≠ E (*P* < 0.005); CAR: F, T0 ≠ E, O (*P* < 0.005, < 0.05); CLI: T0 ≠ E ≠ O (*P* < 0.005)				
B)				
Source	1	11984	2.8389	0.003
Residuals	145	4221.3		
C)				
*S. flabelliformis*				
Light	4	905.26	1.0564	0.35
Time (Light)	5	856.69	0.9997	0.449
Residuals	13	856.95		
*I. basta*				
Light	4	1066.9	0.68538	0.763
Time (Light)	5	1033.4	0.66389	0.805
Residuals	8	1556.6		
*C. coralliophila*				
Light	4	2860.3	1.1666	0.266
Time (Light)	5	1116.7	0.45548	0.998
Residuals	15	2451.8		
*C. foliascens*				
Light	4	1901.4	1.8751	0.001
Time (Light)	5	1105.5	1.0903	0.202
Residuals	18	1014		
Pair-wise Tests				
0 ≠ 0.8, 3.2, 8.1, NL (*P* < 0.005)				
*C. orientalis*				
Light	4	2325.8	1.0689	0.286
Time (Light)	5	3815.6	1.7536	0.001
Residuals	18	2175.9		
Pair-wise Tests				
Within 0 mol photons m^−2^ d^−1^: E ≠ O (*P* = 0.093)				

In pair-wise tests, F: field control, T = 0: time 0 control, E: sampling after the 28 d experimental period, O: sampling after the 14 d observational period; STY for S. *flabelliformis*, IAN for I. *basta*, CYM for *C. coralliophila*, CAR for *C. foliascens* and CLI for *C. orientalis*; 0, 0.8, 3.2, 8.1 mol photons m^−2^ d^−1^ and natural light (3.2–6.5 mol photons m^−2^ d^−1^) within light treatments.
