# Supplementary material for: Effects of light attenuation on the sponge holobiont- implications for dredging management
Source: Sci Rep. 2016 Dec 13;6:39038. doi: 10.1038/srep39038 (PMC5153652; doi:10.1038/srep39038)
Supplement: Supporting Online Material [file srep39038-s1.pdf]

**Supporting Online Material**

# Effects of light attenuation on the sponge holobiont- implications for dredging management

Mari-Carmen Pineda[1,2*], Brian Strehlow[1,2,3], Alan Duckworth[1,2], Jason Doyle[1], Ross Jones[1,2] and Nicole S. Webster[1,2]

[1]*Australian Institute of Marine Science, Townsville, QLD, and Perth, WA, Australia*

[2]*Western Australian Marine Science Institution, Perth, WA, Australia*

[3]*School of Plant Biology and Centre for Microscopy Characterisation and Analysis: University of Western Australia, Perth, WA, Australia*

*Corresponding author:

Mari-Carmen Pineda

*Australian Institute of Marine Science, PMB3, Townsville, QLD, 4810, Australia*

E-mail: mcarmen.pineda@gmail.com.

Tel.: +61 7 4753 4522, fax: +61 7 4772 5852

**Figure S1. Sponges under different light treatments.** Sponge health at the end of the 28 d exposure period, in the phototrophic species (A) *C. orientalis*, (B) *C. foliascens* and (C) *C. coralliophila*, and in the heterotrophic species (D) *S. flabelliformis* and (E) *I. basta*. From left to right, representative sponges exposed to the 0, 0.8, 3.2, 8.1 mol photons $m^{-2}$ $d^{-1}$ treatments and natural light (NL, 3.2–6.5 mol photons $m^{-2}$ $d^{-1}$).

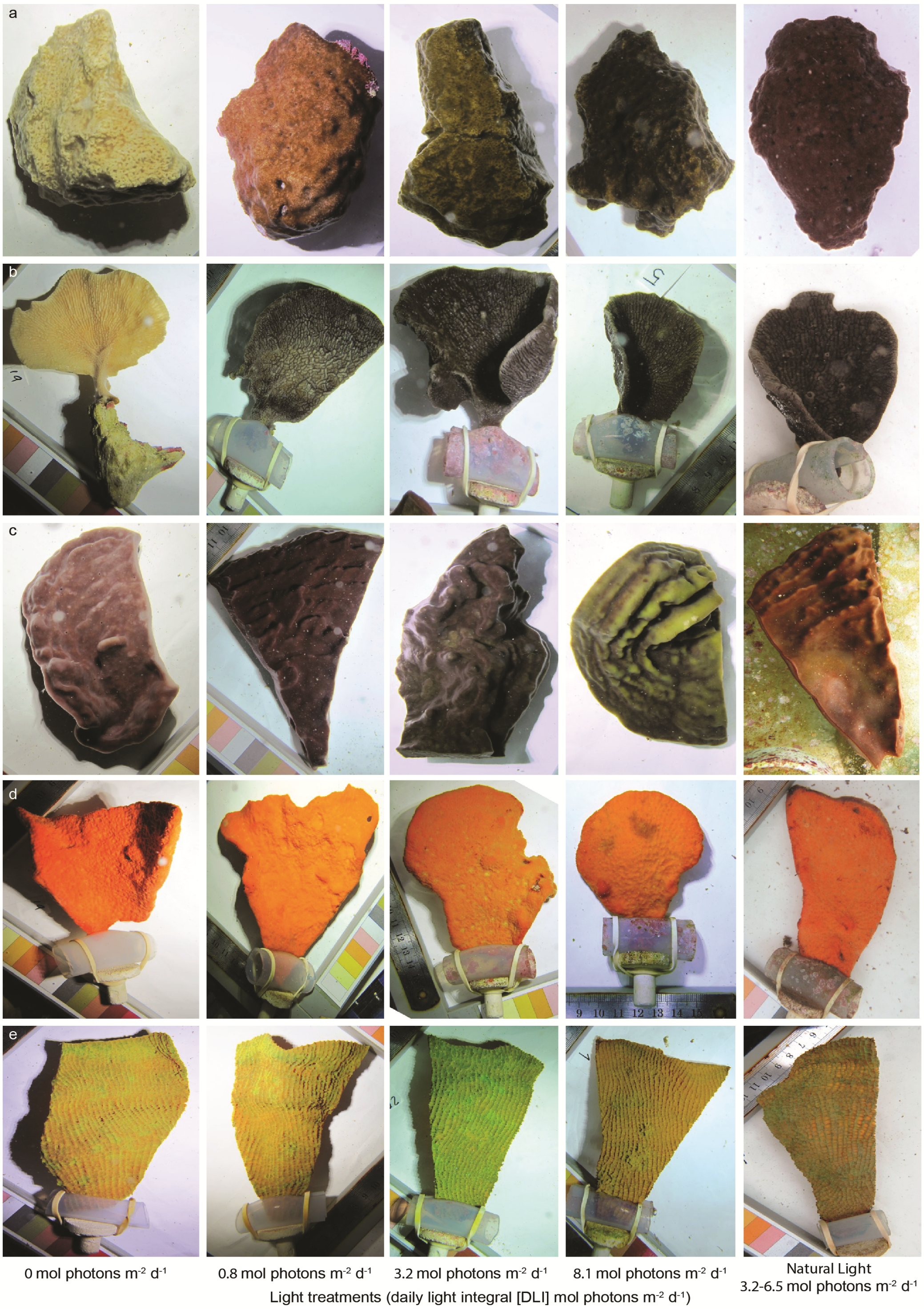

**Figure S2. Area change of all sponges.** Percentage of area change (mean ± SE) for all species and treatments (0, 0.8, 3.2, 8.1 mol photons $m^{-2}$ $d^{-1}$ and natural light (3.2–6.5 mol photons $m^{-2}$ $d^{-1}$)) during the 28 d exposure period and 14 d observational period.

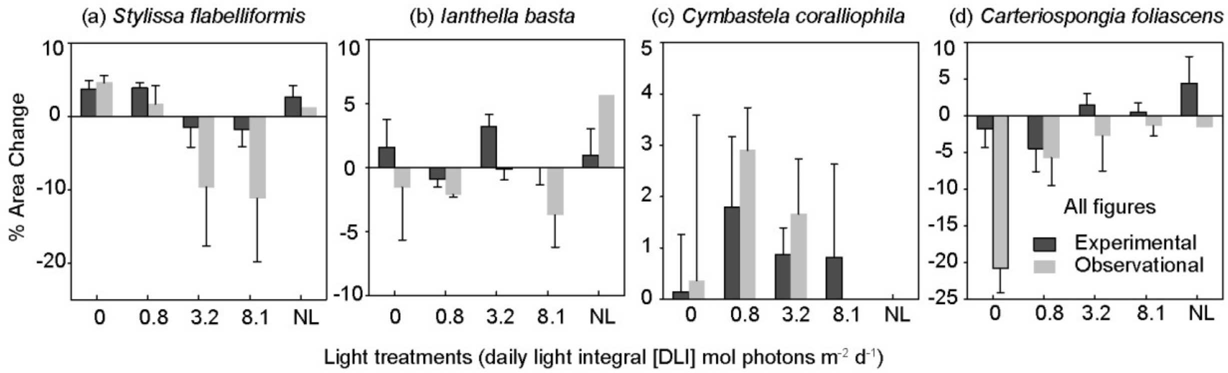


**Figure S3. Non-metric Multi-Dimensional Scaling plots on pigments data.** nMDS of all photopigments retrieved by A) Spectrophotometry (Chl a, Chl b, Chl c, Chl d, Total Chl and Carotenoids) and B) UPLC (Total Chl a, Total Chl, Total Xanthophylls, Total Carotenoids, Chl c2, Peridinin, Neoxanthin, Violaxanthin, Diadinoxanthin, Dinoxanthin, Zeaxanthin, Chl b, Chl a, Chl a epimer, Pheophytin a, α-carotene and ß-carotene). Symbols correspond to species: triangles for *C. coralliophila*, inverted triangles for *C. foliascens*, and squares for *C. orientalis*. Colours represent light treatments: grey for low light intensity treatment (0.8 mol photons $m^{-2}$ $d^{-1}$), black for 0 mol photons $m^{-2}$ $d^{-1}$ and white for natural light control.

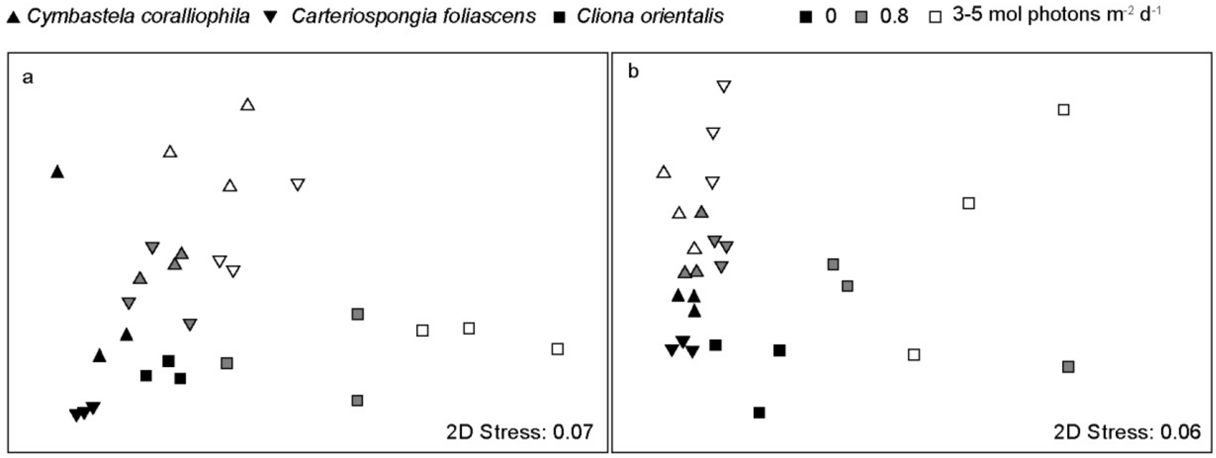

**Figure S4. Non-metric Multi-Dimensional Scaling plots on microbial OTU data.** nMDS of microbial communities for all 5 sponge species and the environmental control (seawater).

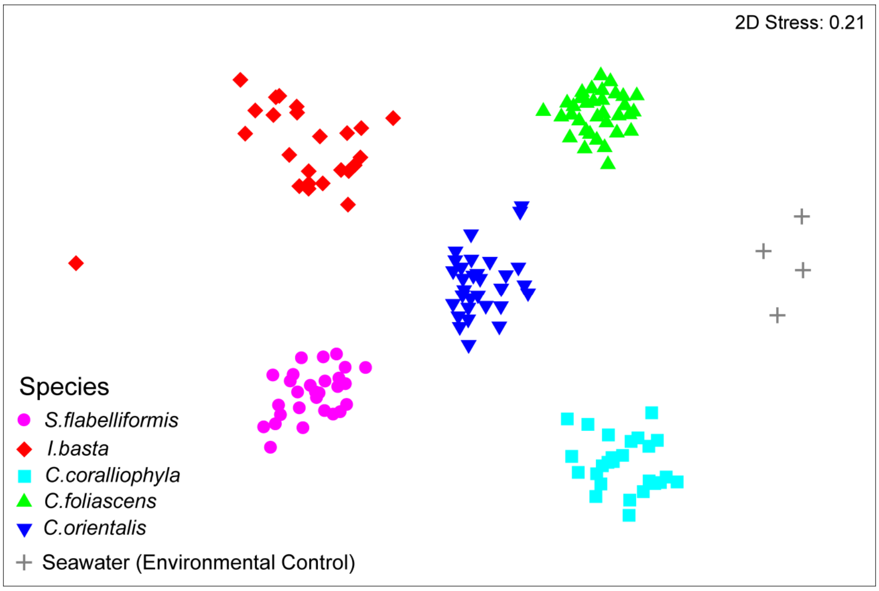

**Table S1. SIMPER results on *C. foliascens* and *C. orientalis*.** Similarity Percentage Analysis (SIMPER) for 30 most significant OTUs driving differences between Natural Light and the 0 mol photons $m^{-2}$ $d^{-1}$ light treatment at 28d and on the species that showed significant differences according to the PERMANOVA analysis (Table 3C).

| OTU | Average relative abundance (%) | | Contribution (%) | Taxonomic ID |
|---|---|---|---|---|
| | Natural Light | 0 | | |
| ***C. foliascens*** | | | | |
| Otu000006 | 5.39 | 0.04 | 6.62 | Cyanobacteria |
| Otu000013 | 3.7 | 1.76 | 2.4 | Gammaproteobacteria |
| Otu000033 | 0 | 1.33 | 1.66 | Alphaproteobacteria |
| Otu000015 | 1.7 | 3 | 1.61 | Gammaproteobacteria |
| Otu000023 | 1.01 | 2.05 | 1.38 | Bacteroidetes |
| Otu000019 | 2.12 | 1.84 | 1.36 | Alphaproteobacteria |
| Otu000034 | 1.25 | 1.84 | 1.34 | Gammaproteobacteria |
| Otu000032 | 0.96 | 2.01 | 1.32 | Gammaproteobacteria |
| Otu000016 | 1.29 | 2.04 | 1.09 | Bacteroidetes |
| Otu000026 | 1.58 | 2.37 | 1.09 | Bacteroidetes |
| Otu000017 | 2.06 | 2.86 | 1.01 | Alphaproteobacteria |
| Otu000059 | 0.29 | 0.75 | 0.81 | Alphaproteobacteria |
| Otu000007 | 4.28 | 4.36 | 0.76 | Bacteroidetes |
| Otu000050 | 0.57 | 0.75 | 0.74 | Gammaproteobacteria |
| Otu000064 | 0.81 | 1.25 | 0.66 | Alphaproteobacteria |
| Otu000048 | 0.68 | 0.82 | 0.63 | Gammaproteobacteria |
| Otu000070 | 0 | 0.51 | 0.63 | Alphaproteobacteria |
| Otu000029 | 1.65 | 2.14 | 0.61 | Gammaproteobacteria |
| Otu000204 | 0.04 | 0.53 | 0.61 | Gammaproteobacteria |
| Otu000073 | 0.37 | 0.36 | 0.6 | Gammaproteobacteria |
| Otu000185 | 0.04 | 0.51 | 0.58 | Deltaproteobacteria |
| Otu000173 | 0.44 | 0 | 0.55 | Cyanobacteria |
| Otu000113 | 0.17 | 0.59 | 0.51 | Chloroflexi |
| Otu000047 | 0 | 0.41 | 0.51 | Bacteroidetes |
| Otu000156 | 0.11 | 0.43 | 0.49 | Gammaproteobacteria |
| Otu000170 | 0.39 | 0 | 0.48 | Gammaproteobacteria |
| Otu000209 | 0.07 | 0.45 | 0.47 | Gammaproteobacteria |
| Otu000077 | 0.24 | 0.28 | 0.44 | Alphaproteobacteria |
| Otu000176 | 0.18 | 0.5 | 0.4 | Gammaproteobacteria |
| Otu000090 | 0.78 | 0.47 | 0.4 | Gammaproteobacteria |
| ***C. orientalis*** | | | | |
| Otu000070 | 0.08 | 1.24 | 1.28 | Alphaproteobacteria |
| Otu000061 | 1.04 | 0 | 1.07 | Cyanobacteria |
| Otu000075 | 0.14 | 1.12 | 1.03 | Gammaproteobacteria |
| Otu000088 | 1.06 | 0.11 | 1.02 | Bacteroidetes |
| Otu000078 | 0.18 | 0.99 | 0.87 | Gammaproteobacteria |
| Otu000083 | 0.1 | 0.88 | 0.84 | Gammaproteobacteria |
| Otu000058 | 0.57 | 1.33 | 0.81 | Unclassified Bacteria |
| Otu000001 | 9.08 | 8.42 | 0.78 | Alphaproteobacteria |
| Otu000104 | 0.7 | 0 | 0.76 | Bacteroidetes |
| Otu000285 | 0.69 | 0 | 0.74 | SBR1093 |
| Otu000067 | 0.11 | 0.65 | 0.65 | Alphaproteobacteria |
| Otu000107 | 0.07 | 0.56 | 0.65 | Alphaproteobacteria |
| Otu000124 | 0 | 0.58 | 0.62 | Alphaproteobacteria |
| Otu000253 | 0.57 | 0 | 0.57 | Alphaproteobacteria |
| Otu000110 | 0.59 | 0.18 | 0.57 | Cyanobacteria |

| | | | | |
|---|---|---|---|---|
| Otu000141 | 0.5 | 0 | 0.52 | SBR1093 |
| Otu000335 | 0.49 | 0 | 0.5 | Gammaproteobacteria |
| Otu000123 | 0.04 | 0.47 | 0.45 | Alphaproteobacteria |
| Otu000091 | 0.17 | 0.57 | 0.43 | Gammaproteobacteria |
| Otu000159 | 0.45 | 0.04 | 0.42 | Alphaproteobacteria |
| Otu000339 | 0.06 | 0.39 | 0.38 | Gammaproteobacteria |
| Otu000286 | 0 | 0.41 | 0.37 | Crenarchaeota |
| Otu000259 | 0.35 | 0 | 0.37 | Alphaproteobacteria |
| Otu000261 | 0 | 0.36 | 0.37 | Chlamydiae |
| Otu000276 | 0.35 | 0 | 0.37 | Alphaproteobacteria |
| Otu000445 | 0.33 | 0.1 | 0.37 | Chloroflexi |
| Otu000273 | 0.04 | 0.37 | 0.36 | Chloroflexi |
| Otu000115 | 0.06 | 0.38 | 0.36 | Alphaproteobacteria |
| Otu000593 | 0.27 | 0.19 | 0.36 | Deltaproteobacteria |
| Otu000079 | 0.36 | 0.04 | 0.35 | Alphaproteobacteria |

**Table S2. Sponge sampling details.** List of species, morphologies, nutritional mode and sampling location.

| **Species Name (Author)** | **Functional Morphology** | **Primary Nutritional Mode** | **Sampling Location** |
|---|---|---|---|
| *Cymbastela coralliophila* (Hooper & Bergquist, 1992) | Encrusting (thick) Cup (table) | Phototrophic[1] | Davies Reef (central Offshore GBR) S 18˚49.354', E147˚38.253′ |
| *Carteriospongia foliascens* (Pallas, 1766) | Cup (wide cup) | Phototrophic[2] | Fantome Island (Palm Islands) S 18°41.028', E 146° 30.706' |
| *Cliona orientalis* (Thiele, 1900) | Encrusting (bioeroding) | Phototrophic[3] | Pelorus Isand (Palm Isands) S 18°32.903', E 146° 29.172' |
| *Stylissa flabelliformis* (Hentschel, 1912) | Erect (laminar) | Heterotrophic[4] | Pelorus Island (Palm Islands) S 18°32.903' E 146°9.172' |
| *Ianthella basta* (Pallas, 1976) | Erect (laminar) | Heterotrophic[5] | Davies Reef S 18˚49.354' 147˚38.253' |

**References**

1. Cheshire, A. *et al.* Preliminary study of the distribution and photophysiology of the temperate phototrophic sponge Cymbastela sp. from South Australia. *Mar. Freshw. Res.* **46,** 1211–1216 (1995).
2. Ridley, C. P., Faulkner, D. & Haygood, M. G. Investigation of Oscillatoria spongeliae-dominated bacterial communities in four dictyoceratid sponges. *Appl. Environ. Microbiol.* **71,** 7366–75 (2005).
3. Schönberg, C. H. L. & Loh, W. K. W. Molecular identity of the unique symbiotic dinoflagellates found in the bioeroding demosponge Cliona orientalis. *Mar. Ecol. Prog. Ser.* **299,** 157–166 (2005).
4. Pineda, M. C., Duckworth, A. & Webster, N. Appearance matters: sedimentation effects on different sponge morphologies. *J. Mar. Biol. Assoc. United Kingdom* **96,** 481–492 (2016).
5. Cheshire, A. C. A., Wilkinson, C. R. C., Seddon, S. & Westphalen, G. Bathymetric and seasonal changes in photosynthesis and respiration of the phototrophic sponge Phyllospongia lamellosa in comparison with respiration by the heterotrophic sponge Ianthella basta on Davies Reef, Great Barrier Reef. *Mar. Freshw. Res.* **48,** 589–599 (1997).
